# Reorientation of Cortical Microtubule Arrays in the Hypocotyl of *Arabidopsis thaliana* Is Induced by the Cell Growth Process and Independent of Auxin Signaling

**DOI:** 10.3390/ijms20133337

**Published:** 2019-07-07

**Authors:** Maciek Adamowski, Lanxin Li, Jiří Friml

**Affiliations:** Institute of Science and Technology Austria (IST Austria), Am Campus 1, 3400 Klosterneuburg, Austria

**Keywords:** microtubules, cortical microtubule arrays, cortical microtubule array reorientation, auxin, cell growth

## Abstract

Cortical microtubule arrays in elongating epidermal cells in both the root and stem of plants have the propensity of dynamic reorientations that are correlated with the activation or inhibition of growth. Factors regulating plant growth, among them the hormone auxin, have been recognized as regulators of microtubule array orientations. Some previous work in the field has aimed at elucidating the causal relationship between cell growth, the signaling of auxin or other growth-regulating factors, and microtubule array reorientations, with various conclusions. Here, we revisit this problem of causality with a comprehensive set of experiments in *Arabidopsis thaliana*, using the now available pharmacological and genetic tools. We use isolated, auxin-depleted hypocotyls, an experimental system allowing for full control of both growth and auxin signaling. We demonstrate that reorientation of microtubules is not directly triggered by an auxin signal during growth activation. Instead, reorientation is triggered by the activation of the growth process itself and is auxin-independent in its nature. We discuss these findings in the context of previous relevant work, including that on the mechanical regulation of microtubule array orientation.

## 1. Introduction

In interphase plant cells, microtubules are found just underneath the plasma membrane. In elongating epidermal cells, such as those in roots, hypocotyls or coleoptiles, these cortical microtubules are aligned into ordered arrays that, especially under the outer plasma membrane, have the ability to conspicuously reorient in relation to the long cell axis. Array orientations have been linked to cell growth status. Put most simply, growing cells typically exhibit microtubules transverse to the long cell axis, and non-growing cells have longitudinally oriented microtubules [1]. However, this simple correlation doesn’t encompass all observed cases. For instance, in light-grown hypocotyls, where elongation is comparatively slow, arrays typically exhibit a continual, slow rotary movement through all orientations, rather than having a stable transverse orientation. A stable transverse orientation is observed in this tissue only during particularly rapid growth phases [2,3,4]. Faster elongating, dark grown hypocotyls also exhibit only limited transverse orientation in the outer epidermal domain [5]. Furthermore, a single cell’s array doesn’t necessarily exhibit a single orientation, but it can be subdivided into domains with microtubules at distinct angles [2].

Because of the general correlation between growth states and microtubule orientations, signals such as light and plant hormones, which are known to regulate growth, have been suggested to mediate reorientation of microtubules as well (summarized in [1]). For instance, the principal growth regulating hormone auxin promotes growth of the hypocotyl and is thought to promote transverse microtubule array orientations in this tissue as well. In the root, a high level of auxin signaling causes an inhibition of growth and instructs the longitudinal orientation of microtubules in the elongation zone. Given such correlations, an early hypothesis stated that reorientation of microtubules by auxin or other factors is in fact a trigger for growth activation or inhibition, a necessary step in the mechanism of growth regulation. This idea was rejected by several studies [6,7], although it was also recently brought to light in the context of root growth inhibition by auxin [8]. Still, other reports have been consistent with this possibility (e.g., [9]).

Alternatively, a more broadly accepted model proposes that growth rate is regulated by mechanisms not involving microtubules. In this scenario, the function for oriented microtubule arrays is not to rapidly regulate the rate of growth but rather to regulate the directionality of growth (degree of anisotropy) in the long term and thus dictate cell and organ shape [10]. The ability of microtubules to control cell wall architecture for this purpose is explained by the so-called alignment hypothesis. It states that microtubules underlying the plasma membrane instruct the movement trajectories of cellulose synthase complexes, which are docked in the plasma membrane and synthesize cellulose fibers in situ. This is possible due to a physical linkage between microtubules and synthase complexes, mediated by CSI1 [11,12,13,14]. Thus, the alignment of newly synthesized cellulose microfibrils in the cell wall would follow the alignment of oriented microtubules underneath [15,16]. By their rigid nature, oriented cellulose microfibrils would then prevent cell expansion in directions that require stretching the fibers while allowing for expansion in directions requiring only the loosening of bonds and increasing distance between adjacent fibers. Interestingly, while the most conspicuous growth-related reorientations are observed in the outer domain of hypocotyl epidermis, it is the relatively stable transverse microtubules in the inner epidermal domain that may contribute more significantly to the anisotropic architecture of cell walls and therefore to hypocotyl organ anisotropy [5].

As mentioned above, the reorientation of microtubules in this model is not a prerequisite for growth activation or inhibition by factors such as auxin or light, but serves only to guide cell shape in the long term. The activation or inhibition of growth would be rather explained by the acid growth theory, encompassing turgor pressure and cell wall rigidity as the competing forces decisive for growth rate. Still, the correlation of the signaling of auxin and other factors, of growth rate changes, and of array reorientations, has led researchers to suggest that auxin, light, and other similar factors not only regulate growth but also simultaneously act as triggers for microtubule reorientation.

However, a different chain of causality can be proposed instead, one where these factors only trigger signaling leading to the regulation of growth, while the associated reorientations of microtubules are downstream consequences of growth activation or inhibition but are not directly controlled by hormonal or light signaling pathways. Previously, such considerations were put to test with experiments in maize coleoptiles, azuki bean epicotyls, and sunflower hypocotyls. As models representing differential growth, these experiments involved the mechanical stretching or bending of these plant organs or epidermal tissue isolates. For instance, Fisher and Schopfer [17] tested whether microtubules on the two sides of a tropically bending maize coleoptile react to gravitropic and phototropic stimuli (which implies direct regulation by light and/or auxin signaling) or rather to the differential cell deformation (elongation) that is associated with tropic growth. To uncouple these two potential factors, coleoptiles were forcibly bent in a direction opposite to that promoted by a simultaneously applied tropic stimulus. Cortical microtubules aligned according to the imposed deformation of the organ and not according to the ‘opposing’ tropic stimulus. This result indicates that it is the actual change of cell shape that determines microtubule array orientation—not the signaling activity of auxin or light. A similar experiment was performed on azuki bean epicotyls with comparable results [18].

In contrast to this, Himmelspach and Nick [19] argued that microtubules reorient in response to a gravitropic stimulus, rather than in response to growth changes. Furthermore, Takesue and Shibaoka [9] showed that auxin-treated azuki bean epicotyl segments exhibit transverse microtubules even when their growth is inhibited by anaerobic conditions, demonstrating the ability of auxin to cause reorientations independently of growth and, thus, also arguing against the idea that it is growth induced by auxin which causes reorientation of microtubules. Finally, Burian and Hejnowicz attempted to elicit cell deformation-dependent reorientations in peels of epidermis isolated from sunflower hypocotyls and stretched by external forces. However, in this experimental system, cell deformation was insufficient to induce reorientation [20], unless fusicoccin, a growth-activating drug acting similarly to auxin by the activation of proton pumps and potassium channels, was additionally applied [21].

As can be seen from all of the above, the relationship between auxin, the activation or inhibition of growth, and the reorientation of cortical microtubule arrays has been a matter of debate. Here, we revisit this problem with a comprehensive, well-controlled set of experiments performed in the model plant *Arabidopsis thaliana* (hereafter *A. thaliana*). We study the processes in elongating cells of the hypocotyl, during the promotion of growth by auxin which is associated with the reorientation of microtubules into transverse arrays. We address the problem in a direct way, omitting experimentally imposed mechanical bending or stretching as proxies for growth, as well as tropic responses, in favor of controlling normal organ growth and auxin signaling with the now available chemical and genetic tools. Our study is specifically aimed at addressing the role of auxin in cortical microtubule array reorientation, while it does not address the mode of action of other hormones, or light, on the microtubule cytoskeleton.

## 2. Results

### 2.1. Hypotheses Relating Auxin Signaling, Growth Promotion, and Transverse Microtubule Array Reorientation in the Hypocotyl

**Figure 1:**
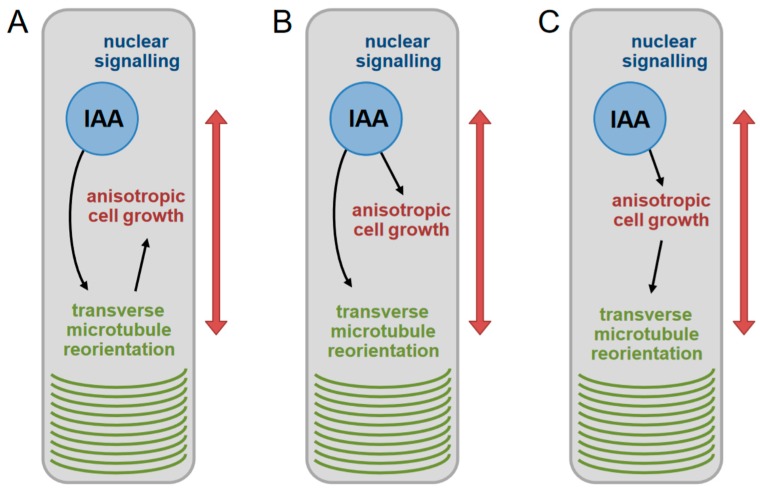
Hypotheses describing the relationship between auxin signaling, cell growth, and cortical microtubule reorientations in elongating hypocotyl cells. (**A**) Hypothesis A: Auxin triggers growth through reorienting microtubules into transverse arrays. The reorientation by auxin is a necessary part of the mechanism by which growth is activated. (**B**) Hypothesis B: Auxin triggers cell growth and the reorientation of microtubules by two parallel signaling mechanisms. Both these auxin signaling branches are activated in elongating cells of the hypocotyl. (**C**) Hypothesis C: Auxin triggers only cell growth, while the reorientation of microtubules is a downstream consequence of anisotropic growth and is by itself auxin independent.

To formalize the research problem, we defined and tested three competing hypotheses describing the relationship between auxin signaling, the promotion of growth, and the transverse reorientation of cortical microtubules (Figure 1). These three models encompass various scenarios previously considered in the literature and outlined above. In the first scenario (Hypothesis A, Figure 1A), auxin directly triggers reorientation of microtubules, and this reorientation is a necessary part of a mechanism by which auxin promotes growth. While this model has been rejected by the majority of previous studies, we include it for clarity and completeness of analysis. In contrast, in the remaining two scenarios, growth is activated by a mechanism not requiring the reorientation of microtubules. Here, reorientation, is a phenomenon associated with growth activation for other purposes. Hypotheses B and C differ in regard to the direct trigger of reorientation. In Hypothesis B (Figure 1B), it is auxin signaling that triggers the reorientation of microtubules simultaneously with triggering growth by another branch of its signaling pathways. These two signaling branches of auxin are temporally correlated during normal growth but are in principle separable. This hypothesis, while not necessarily formulated precisely in these terms, refers to models describing auxin as able to reorient microtubules, while recognizing that growth activation by auxin is explained by the acid growth theory or other mechanisms not involving microtubules. Finally, under Hypothesis C (Figure 1C), only growth is directly activated by auxin signaling, while the reorientation of microtubules into transverse arrays happens as a downstream consequence of the anisotropic cell growth. The reorientation signal comes from the growth itself and is, in principle, auxin independent. Among the three hypotheses outlined above, our experiments suggest Hypothesis C as most closely describing the causal chain involving auxin signaling, microtubule array reorientation, and growth, as will be demonstrated below.

### 2.2. Auxin-Induced Reorientation of Cortical Microtubules in Isolated Hypocotyls of A. thaliana

**Figure 2:**
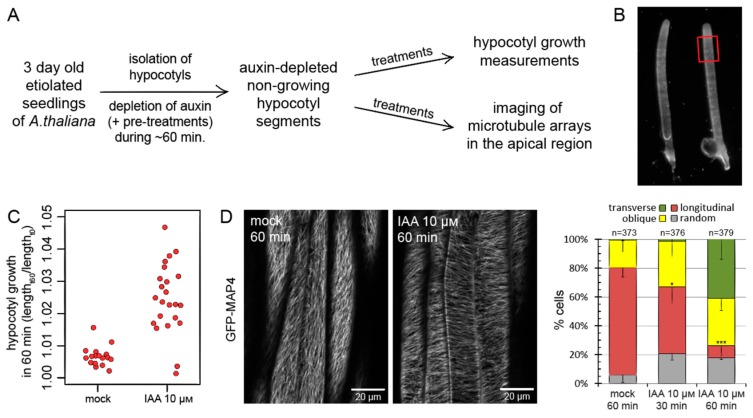
Auxin-induced reorientation of cortical microtubules in isolated hypocotyl segments of *A. thaliana*. (**A**) An overview of the experimental setup used throughout this study. Hypocotyls were isolated from three-day-old etiolated seedlings of *A. thaliana*. The depletion of endogenous auxin and the resulting cessation of growth was allowed within following time of around one hour. Necessary chemical pre-treatments were applied in this period. Hypocotyls were then transferred to treatment media (e.g., containing auxin) and imaged by CLSM typically after one hour of treatments, or they were placed on a flatbed scanner for growth measurements. See Section 4 for details. (**B**) Isolated *A. thaliana* hypocotyls used in this study. Red rectangle marks the sub-apical region of active growth in three-day-old hypocotyls, where the orientations of microtubules were scored. (**C**) Auxin-induced growth of isolated *A. thaliana* hypocotyls. Auxin-depleted hypocotyls were placed on control or auxin-supplemented (IAA, 10 μM) media, and their growth was measured over a one hour period. The graph shows collated growth measurements from three experiments, each data point representing one hypocotyl. (**D**) Concomitant with growth, auxin (IAA, 10 μM) causes the reorientation of cortical microtubule arrays in the outer faces of hypocotyl epidermis, from predominantly longitudinal into predominantly oblique and transverse. Cells were scored into four categories based on the prevalent type of microtubule array observed. The GFP fluorescence is depicted in grayscale. The graph shows average percentage of cells in each category from three experiments. Error bars indicate standard deviation (only the lower bar is shown for clarity). Total cell numbers are given above the graph. The frequencies of cells with longitudinal arrays were compared with the mock treatment using *t* tests, *p* = 0.022 (*) for 30 min auxin treatment and *p* = 0.0003 (***) for 60 min auxin treatment.

Throughout this study, we used three-day-old, dark-grown hypocotyls of *A. thaliana* which were separated from the apical part of the seedling and from the root (Figure 2A,B; [22,23]). After separation from the apical auxin source, hypocotyls become depleted of endogenous auxin and cease growth within approximately 30 min [22,23]. Consequently, growth and auxin signaling, two factors under consideration, can be controlled by the external application of auxin. The use of dark-grown seedlings aids in achieving highly anisotropic and rapid growth. To visualize cortical microtubule arrays, we used confocal laser scanning microscopy (CLSM) with the *35S:GFP-MAP4 (GREEN FLUORESCENT PROTEIN-MICROTUBULE ASSOCIATED PROTEIN 4)* microtubule marker line. We imaged microtubules in the outer domain of the epidermis in the sub-apical region of the hypocotyl, where rapid growth occurs in three-day-old etiolated seedlings (red rectangle in Figure 2B). In each repetition of a single experiment, 8–16 hypocotyls and around 50–100 cells were scored for each condition or genotype. Since the reorientation processes are inherently variable, our approach was to compare the end-point microtubule orientations on large populations of hypocotyls and cells in order to derive the typical response rather than to follow the dynamic microtubule behavior over time in single cells at the cost of sample size.

For the scoring of microtubule orientation, we categorized cells according to a previously established manner into ‘longitudinal,’ ‘oblique,’ ‘transverse,’ and ‘random’ classes, based on the predominant orientation of microtubules in relation to the long cell axis. The ‘random’ class refers to cells where microtubules were disordered or in which multiple orientations could be observed with a similar contribution. Microscopic pictures taken at all conditions or from all genotypes in a single experiment were combined together, randomized by a computer script, and then scored without explicit knowledge of the condition at which each particular picture was taken. An automated quantification of microtubule orientation was not employed, as the observed changes in orientation were of large magnitude and could be safely evaluated by the above-described method.

In auxin-depleted, non-growing hypocotyls, cortical microtubules were predominantly longitudinal, in agreement with established knowledge (Figure 2D). As expected, the application of auxin (10 μM of indole-3-acetic acid or IAA) triggered rapid growth of hypocotyl segments (Figure 2C; [22,23]) and triggered oblique and transverse arrays, with clear reorientation visible after one hour of treatment (Figure 2D).

### 2.3. Auxin Mediates Microtubule Array Reorientation by the Transcriptional Pathway

The classical, transcriptional signaling pathway of auxin (reviewed in [24,25]) involves the perception of auxin in the nucleus by the TIR1/AFB (transport inhibitor response 1/auxin signaling f-box protein) receptors, which are part of the SCF^TIR1/AFB^ (Skp1-Cullin-F-box) E3 ubiquitin ligase complex. Prior to auxin perception, proteins of the Aux/IAA (auxin/indole-3-acetic acid) class dimerize with and act as repressors of the ARF (auxin response factor) family of transcription factors. As a result of auxin perception, Aux/IAAs are ubiquitylated by the ubiquitin ligase complex and degraded in the proteasome. ARFs are released from their inhibited state and activate transcription from genes containing auxin-responsive elements (AuxREs) in promoter sequences.

**Figure 3:**
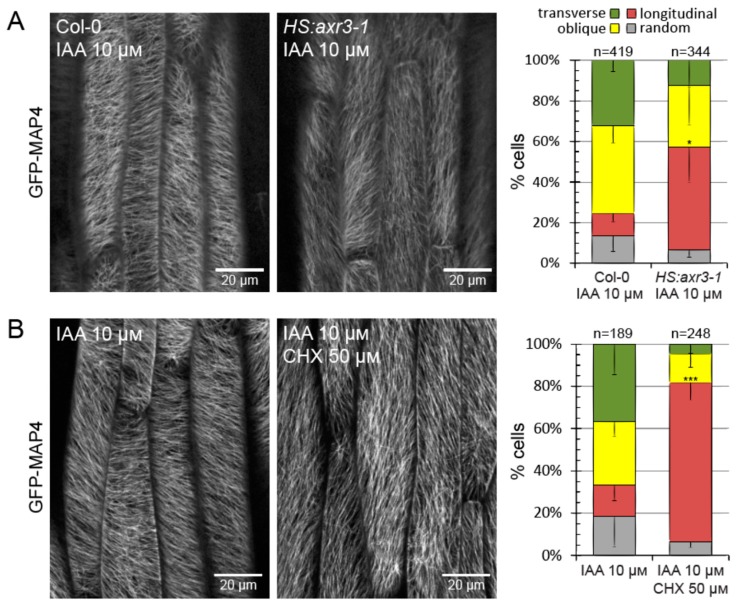
Cortical microtubule array reorientation by auxin is mediated by the SCF^TIR1/AFB^-Aux/IAA-ARF nuclear signaling pathway. The inhibition of nuclear auxin signaling by the conditional expression of a dominant-negative mutant of the Aux/IAA protein AXR3/IAA17 (*HS:axr3-1*) (**A**) or by blocking protein translation with cycloheximide (CHX, 50 μM) (**B**) prevented the auxin-induced reorientation of cortical microtubules. The GFP fluorescence is depicted in grayscale. The graphs show the average percentage of cells in each category from three experiments. Error bars indicate standard deviation (only the lower bar is shown for clarity). Total cell numbers are given above each graph. The frequencies of cells with longitudinal arrays were compared using *t* tests, *p* = 0.0176 (*) in **A** and *p* = 0.0007 (***) in **B**.

Recently, it was demonstrated that hypocotyl growth is activated by auxin via the SCF^TIR1/AFB^-Aux/IAA-ARF signaling pathway [23]. We tested the involvement of this signaling pathway in the growth-associated reorientation of microtubules by the use of axr3-1, a mutant variant of the Aux/IAA protein AXR3/IAA17 which is not degraded following auxin perception [26,27] and continuously represses auxin response in the hypocotyl. The expression of axr3-1 was induced from a heat-shock promoter (*HS:axr3-1*) approximately three hours before starting the experiment. We also used the drug cycloheximide (CHX), which inhibits protein translation, blocking the auxin-responsive gene activation at the level of protein biosynthesis as well. In both these conditions, auxin is unable to induce growth in isolated hypocotyls [23]. With both these interferences, we also abolished the auxin-induced microtubule reorientation (Figure 3). This shows that in the hypocotyl, both responses to auxin: Growth [23] and microtubule array reorientation occur through the SCF^TIR1/AFB^-Aux/IAA-ARF signaling pathway.

### 2.4. An Intact Microtubule Array Is Not Required for Auxin-Mediated Activation of Hypocotyl Growth 

Having established this, we turn to the three hypotheses causally relating auxin signaling, growth, and microtubule reorientation. We first addressed the scenario in which auxin signaling activates growth through an obligatory step of microtubule array reorientation (Hypothesis A). A prediction of this hypothesis is that, following disruption of microtubules, auxin will be unable to trigger hypocotyl growth. In contrast, the remaining two hypotheses predict that auxin will be able to trigger growth even in the absence of functional microtubules.

**Figure 4:**
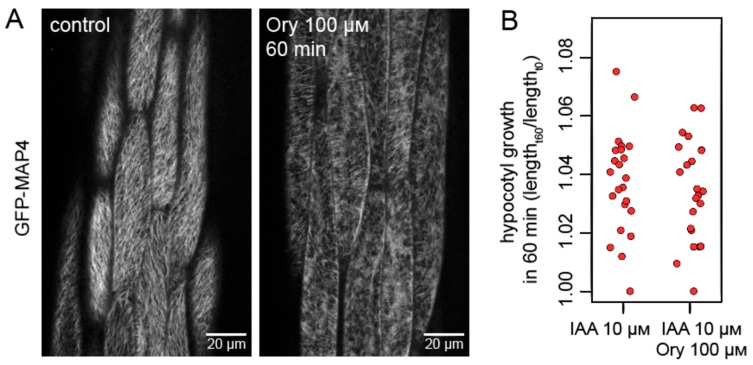
Intact microtubule arrays are not necessary for the promotion of hypocotyl growth by auxin. (**A**) The effect of oryzalin in *A. thaliana* hypocotyl segments. A majority of microtubules are disrupted after 60 min of oryzalin (100 μM) treatment. The GFP fluorescence is depicted in grayscale. (**B**) Elongation of isolated hypocotyls during one hour of auxin treatment (IAA, 10 μM) after the disruption of microtubules by oryzalin (100 μM). Oryzalin was applied as a pre-treatment for 60 min. as well as during the subsequent auxin treatment. The disruption of microtubules did not interfere with auxin-induced growth. The graph shows collated growth measurements from three experiments, each data point representing one hypocotyl. Hypocotyl growth rates were compared using a *t* test, *p* = 0.6593.

We first pre-treated hypocotyls with the microtubule disrupting drug oryzalin for one hour and then applied oryzalin with auxin and monitored growth. We used oryzalin at a high concentration (100 μM) which typically caused the depolymerization of a majority of microtubules (Figure 4A). Some microtubules were retained, possibly due to a stabilizing activity of the MAP4 protein itself [28]. We found that the disruption of most of the microtubules did not interfere with auxin-induced growth during a one hour growth period (Figure 4B). This result supports the notion that microtubule reorientation is not an essential part of the mechanism by which auxin activates growth.

### 2.5. Microtubule Array Reorientation Triggered by Auxin Requires the Presence of Growth

Next, we considered Hypotheses B and C. Here, growth is activated by a mechanism not involving microtubules. Our aim was to distinguish whether the reorientation of microtubules is mediated by auxin signaling directly, along with the activation of growth by auxin, by two branches of SCF^TIR1/AFB^-Aux/IAA-ARF signaling, or, alternatively, whether the reorientation of microtubules is an indirect consequence of auxin-triggered growth, not directly controlled by a branch of auxin signaling. To this end, we attempted to limit or inhibit the growth of hypocotyl segments while maintaining high auxin signaling. Under Hypothesis B, microtubules will be equally reoriented because their orientation responds to auxin signaling directly, while Hypothesis C predicts that microtubules will not reorient properly because their orientation responds to growth rather than to auxin signaling.

**Figure 5:**
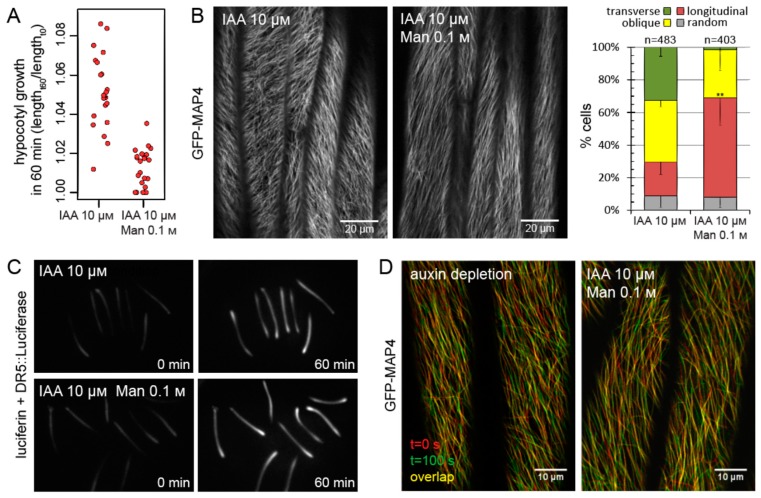
Cortical microtubule array reorientation triggered by auxin depends on growth. (**A**) Reduction in rates of auxin-induced growth of hypocotyl segments by increased osmolarity. 0.1 M of mannitol (Man) was added to the medium to counteract water uptake and turgor increase. The graph shows collated growth measurements from three experiments, each data point representing one hypocotyl. (**B**) Auxin-induced reorientation of microtubules into transverse arrays depends on growth. In auxin-treated hypocotyls which are prevented from growing rapidly by 0.1 M of mannitol, microtubules do not reorient towards transverse arrays efficiently after a one hour growth period. The GFP fluorescence is depicted in grayscale. The graph shows average percentage of cells in each category from four experiments. Error bars indicate standard deviation (only the lower bar is shown for clarity). Total cell numbers are given above the graph. The frequencies of cells with longitudinal arrays were compared using a *t* test, *p* = 0.0045 (**). (**C**) Control for auxin signaling. 0.1 M of mannitol did not interfere with auxin signaling in hypocotyl segments, as visualized using luciferin and the *DR5:Luciferase* auxin signaling reporter line. (**D**) Control for microtubule dynamics. In hypocotyls treated with 0.1 M of mannitol, microtubules exhibited normal signs of assembly and disassembly. Longitudinal arrays after combined IAA and mannitol treatment are compared with similar longitudinal arrays in auxin depleted hypocotyls. Two pictures taken 100 s apart are overlapped. The overlapping fraction of microtubules is shown in yellow, while red and green shows microtubules that differ, demonstrating the occurring polymerizations and depolymerizations. The figure is representative for 39 movies captured per treatment condition.

Plant cell growth is a result of the hydrostatic pressure of the vacuole and cytoplasm, called turgor, which acts to expand the cell by overcoming the mechanical resistance of the encompassing cell wall. To reduce hypocotyl growth, we mildly increased the osmotic conditions by adding 0.1 M of mannitol to the medium in order to diminish the build-up of turgor. Indeed, after auxin treatment, hypocotyls placed on a mannitol-supplemented medium grew less than control hypocotyls (Figure 5A). In these hypocotyls, microtubules did not reorient into transverse arrays as efficiently as in rapidly growing controls, instead retaining mostly longitudinal orientations (Figure 5B). As a control, using luciferin and *DR5:Luciferase*, a line expressing the enzyme luciferase under the control of an auxin-responsive promoter, we verified that 0.1 M of mannitol does not block auxin signaling (Figure 5C). Furthermore, by time-lapse imaging of GFP-MAP4, we observed that with 0.1 M of mannitol, the microtubules still exhibited normal signs of assembly and disassembly, necessary for the reorientation of the array (Figure 5D). Thus, even though auxin signaling was high and microtubules retained their activities, the array did not reorient efficiently when cell growth was reduced. In other words, the degree of array reorientation correlated with cell growth rate but did not correlate with auxin signaling levels. This suggests that microtubules in the epidermis of hypocotyls may, in fact, not respond to auxin signaling; rather, they specifically respond to growth.

### 2.6. Growth without Auxin Signaling Is Sufficient to Reorient Microtubule Arrays

Therefore, we next wanted to activate hypocotyl segment growth in the absence of auxin. For that purpose, we used fusicoccin (FC), a toxin of the plant pathogenic fungus *Fusicoccum amygdali* that has an auxin-like effect specifically on cell growth while not acting as auxin in the sense of binding to the auxin receptor and triggering nuclear signaling. Fusicoccin triggers cell growth by at least two mechanisms consistent with the acid growth theory of auxin action. First, it activates the plasma membrane proton pumps of the AHA (autoinhibited H^+^-ATPase) family by physically binding their C-termini with the regulatory 14-3-3 proteins [29]. This mimics proton pump activation during normal auxin response, mediated by at least some of the early auxin-inducible *SAUR* (*SMALL AUXIN UP RNA*) genes and by proton pump phosphorylation [22,30,31]. Additionally, fusicoccin binds to and activates the inward rectifying K^+^ channels while deactivating the outward rectifying K^+^ channels [32,33], leading to an increase in internal K^+^ concentration, which promotes water uptake and turgor build-up. This activity further parallels the physiological growth mechanism.

**Figure 6:**
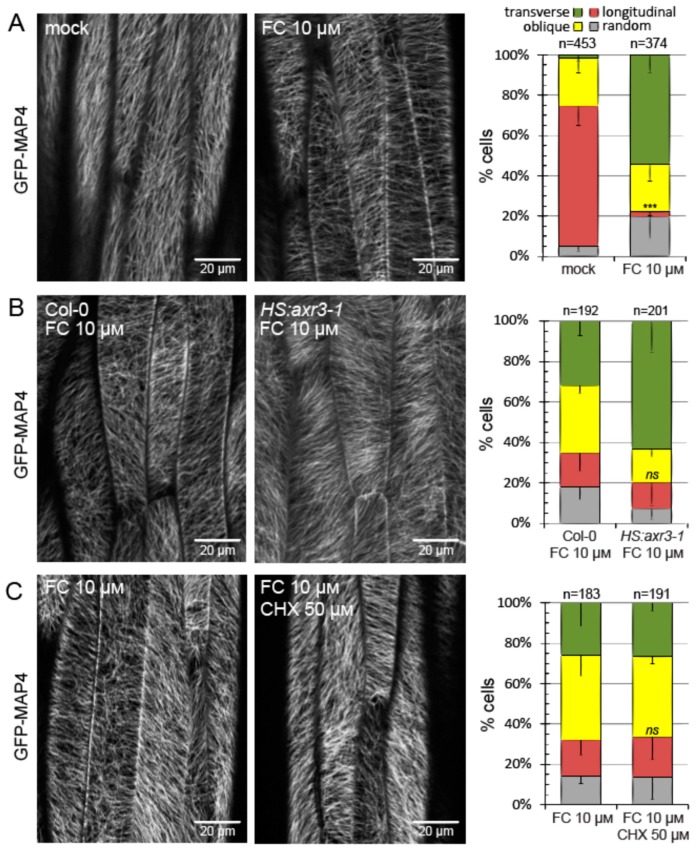
Fusicoccin-induced growth causes microtubule array reorientation in the absence of auxin signaling. (**A**) Auxin depleted hypocotyl segments induced to grow with fusicoccin (FC, 10 μM) for one hour exhibit transversely oriented microtubule arrays. (**B**,**C**) Additional controls ascertaining auxin-independence of the effect. Fusicoccin can induce transverse microtubule array reorientation in the growing hypocotyls of the auxin signaling mutant *HS:axr3-1* and following the inhibition of protein translation by cycloheximide (CHX, 50 μM). The GFP fluorescence is depicted in grayscale. The graph shows average percentage of cells in each category from three experiments. Error bars indicate standard deviation (only the lower bar is shown for clarity). Total cell numbers are given above each graph. The frequencies of cells with longitudinal arrays were compared using *t* tests, *p* = 0.0003 (***) in **A**, *p* = 0.6569 in **B** and *p* = 0.813 in **C**. ns—not significant.

Fusicoccin caused a rapid elongation of auxin-depleted hypocotyl segments, as previously demonstrated [23]. After one hour of this treatment, microtubules were found to be reoriented into transverse and oblique arrays (Figure 6A). This directly demonstrates that cell growth can trigger microtubule reorientation independently of auxin. While the isolated hypocotyl segments are depleted of endogenous auxin, and as expected, fusicoccin does not trigger auxin signaling in them [23], we additionally ascertained the auxin independence of the effect by two control experiments. Namely, fusicoccin could still trigger reorientation when auxin signaling was blocked by expressing axr3-1 or when all protein translation—including that necessary for auxin-induced gene expression—was blocked by cycloheximide (Figure 6B,C). In both these conditions, fusicoccin was effective at inducing hypocotyl segment growth, contrary to auxin [23], because the direct growth activation by fusicoccin bypassed nuclear auxin signaling.

In summary, our results collectively argue for Hypothesis C (Figure 1C), where the transverse reorientation of microtubules is a consequence of growth but by itself is auxin-independent. As an illustration to this, the results presented in Figure 3 can be now correctly interpreted: Interference with the SCF^TIR1/AFB^-Aux/IAA-ARF signaling pathway prevented microtubule reorientation by auxin only indirectly, because auxin was unable to activate growth.

## 3. Discussion

### 3.1. The Auxin Effect on Cortical Microtubule Orientation in the Hypocotyl is Indirect and Explicable by Underlying Cell Growth

This work investigated the cause of transverse cortical microtubule array reorientation in the outer face of epidermis in growing hypocotyls. We used hypocotyl segments isolated from the apical and basal parts of the seedling, thanks to which we gained experimental control of growth and of auxin presence. While in an intact plant, there may exist systemic signals influencing microtubule arrays of the hypocotyl, the observation that reorientations were possible and followed predictable patterns evidences that the isolated hypocotyl contains all necessary controls for microtubule reorientations, and, thus, that the experimental system well captured the fundamental behaviors of cortical microtubule arrays.

We first showed that auxin triggers the transverse reorientation of microtubules through the SCF^TIR1/AFB^-Aux/IAA-ARF signaling pathway (Figure 3), just like it triggers cell growth [23]. We confirmed the notion that the reorientation of microtubules (or the microtubule array as a whole) is not necessary for growth activation by auxin (Figure 4). The transverse reorientation of microtubules only coincides with, but does not cause, cell growth. Then, we asked if, in fact, reorientation is triggered by SCF^TIR1/AFB^-Aux/IAA-ARF auxin signaling while it also triggers growth, or whether reorientation is triggered by growth while not strictly requiring auxin. By uncoupling the two phenomena that normally coexist in growing hypocotyls, auxin signaling and cell growth, we showed that activation of auxin signaling is not sufficient for reorientation if growth is diminished by reducing turgor build-up (Figure 5) and that the activation of growth in the absence of auxin signaling is sufficient to trigger reorientation (Figure 6). Thus, we concluded that the SCF^TIR1/AFB^-Aux/IAA-ARF auxin signaling pathway is not directly responsible for controlling microtubule arrays.

Our data show that the signaling for microtubule reorientation originates from growth. However, it has been previously reported that auxin can induce the transverse reorientation of microtubules in azuki bean epicotyls that are inhibited from elongation by an anaerobic environment [9]. With our demonstration of reorientations in auxin-free conditions, we can anticipate that the same effect could have been achieved with fusicoccin. Still, since anaerobic conditions blocked elongation [9], the result of Takesue and Shibaoka is still contradictory to a simple conclusion about cell elongation as a reorientation trigger. Along the same lines, the transverse reorientation of microtubules has been observed after fusicoccin treatments in epidermis peels isolated from sunflower seedling hypocotyls, while under this treatment, no elongation of these tissue isolates was reported [21]. The above examples suggest that the trigger for reorientation could lie somewhere in the cellular mechanism of growth while not necessarily requiring elongation itself. The trigger could be related to some properties of the cell interior (turgor) and/or exterior (mechanical properties of the cell wall, discussed below) that could be separable from, and only approximately correlated with, the rate of cell elongation itself. As such, while throughout this work we used the term ‘growth,’ perhaps a more accurate term to describe the cause of microtubule reorientation could be a broadly defined ‘growth process’ or a ‘physiological condition of the cell associated with growth’ rather than growth understood strictly as cell elongation. In our experiments, we were able to activate reorientation with fusicoccin, which affects plasma membrane proton pumps and potassium channels and therefore influences external pH and cell wall properties in addition to promoting water uptake to increase turgor. In turn, we were able to block the reorientation trigger by the use of mannitol, which presumably reduced water uptake and turgor increase.

Our findings may aid in the interpretation of various experimental observations related to microtubule array orientation. For instance, it was recently observed that the inhibition of auxin-induced maize coleoptile segment growth by naphthazarin, a naturally occurring plant secondary metabolite, also reduced the degree of auxin-induced microtubule array reorientation [34]. Most likely, the effect of naphthazarin is explainable by the underlying changes to cell growth or some related aspects of cell physiology.

While our study excluded a direct effect of auxin signaling on microtubule array reorientation in elongating hypocotyl cells, we do not make inferences about the mode of action of other hormones, or light, on cortical microtubule arrays. These other factors may either act through the modulation of the growth process or may have a more direct influence on microtubule dynamics.

### 3.2. Microtubule Array Control by Mechanical Forces and Its Connection to Growth

As concluded above, microtubule array reorientation is triggered by changes associated with cell growth, likely related to turgor and to the cell wall. Cell walls, as load-bearing elements of the cell, experience tension in reaction to the turgor pressure of the cytoplasm [35]. On a larger scale, whole tissues experience stresses, which can be either tension or compression, as a result of the influence of other tissues. For instance, it is thought that in the stem, the outer tissues are under constant tension, while the inner tissues under constant compression. The opposite stresses experienced by these two parts of the organ result from being firmly attached to the other part which “wants to” grow at a different rate [36,37].

Among previously published work on cortical microtubules, a prominent idea describes stresses acting within cell walls as factors regulating microtubule array orientation. The concept that these stresses can influence microtubules in elongating epidermal cells was explored through experiments involving the bending of hypocotyls and coleoptiles or by applying forces to isolated peels of epidermis. Indeed, in certain conditions, it can be observed that cells on the stretched, outwardly bulged side of a mechanically bent organ exhibit transverse arrays, like elongating cells do, while compressed cells on the inwardly curved side can exhibit longitudinal arrays, like cells that do not elongate [1,17]. Such observations can be translated to normal physiological growth, the idea being that experimentally imposed bending produces tensions and compressions, and changes of cell dimensions, that imitate natural growth and the cell wall stresses associated with it. For this reason, some of the work that used externally applied forces arrived at conclusions similar to the ones reached here by us with a direct experimentation on growth [17]. That said, cases have also been reported where the mechanical extension of tissues induced longitudinal, rather than transverse, microtubule orientations [38,39,40]. Therefore, it is unclear whether experimentally applied mechanical manipulations of cells or tissues can be always safely interpreted as representing naturally occurring anisotropic growth.

Going further, both types of wall stress—tension and compression—may be anisotropic—that is, act predominantly along a particular direction in plane of the cell wall. This could be due to specific geometries of cells and their tissue environment, as well as the anisotropic architecture of the load-bearing cellulose mesh in the cell wall. Further exploring the idea of forces controlling microtubule array orientations, Hamant and collaborators proposed that in the epidermis, the orientations of predominant tension in the outer cell wall instruct similar orientations for underlying microtubules, both when considered at a single cell level and on a broader scale of the epidermal tissue as a whole [41]. This model was first proposed for the epidermis of the shoot apical meristem, a dome-shaped structure with bulging, growing organ primordia. The model is based on the comparison between microtubule orientations observed on the meristem surface and modelled (predicted) wall stresses (specifically, tensions). Further evidences include experiments with cell ablations and the compression of the meristem. The same mechanism was proposed to function in the puzzle piece-shaped epidermis of the leaf [42]. Among other evidence, it can be seen that large scale ablations in the leaf, which change the distribution of tensions in the adjacent tissue, produce a pattern of synchronized microtubule orientations that are aligned with the predicted tensions.

Recently, Robinson and Kuhlemeier produced a transverse reorientation of microtubules in the outer domain of hypocotyl epidermis by applying compression to the hypocotyl along its long axis [40]. The modelling of tissue tensions predicted that such compression of the hypocotyl produces circumferential tension in the outermost cell walls of the hypocotyl, which corresponds with transverse microtubule orientation along the lines of the model outlined above. Thus, it appears that the orientation of microtubules according to cell wall tension directions can in principle occur in the epidermis of the hypocotyl. It becomes interesting to consider whether a directional tension-based mechanism can also explain the longitudinal-transverse orientation changes related to the growth status of the hypocotyl. If this is the case, changes in the growth status would have to be associated with a dramatic switch of predominant tissue tension in the epidermis, between a circumferential tension during growth and axial tension when growth has ceased. Thus far, scarce experimental evidence, as well as theoretical considerations, have suggested that cell wall tension on the stem surface is predominantly axial [40,43]. It may be perhaps conceived that during rapid growth, axial tension is relaxed while circumferential tension persists, and, by that process, transverse microtubule orientation is instructed. In fact, observations of longitudinally split hypocotyl segments which were isolated from tropically responding plants support the notion that rapidly elongating stem tissue may be losing axial tension [37].

Apart from the lack of systematic data on the distribution of tensions in stems in relation to the growth status, there is still another difficulty with adapting to stem epidermis the concept of microtubule orientation regulation by the directionality of cell wall tensions. Cell wall tensions are described as acting on a broad scale, being distributed and synchronized on the tissue scale rather than varied from cell to cell. In contrast, microtubule arrays can have different orientations in neighboring cells, and, as mentioned in the introduction, can in some instances exhibit continual rotary motions during growth rather than having more-or-less stable orientations [2,3]. These appear particularly difficult to reconcile with models involving microtubule orientation control by tissue tension.

To sum up the above, the possibility of a mechanical influence on microtubule array orientations has been unequivocally demonstrated in experiments, but it is not exactly clear how mechanics-based microtubule control could work during growth-related reorientation in stems, because it is also not clear which changes to distribution of tensions in the outer cell walls are associated with the changing growth status in this type of organ.

Interestingly, some of the experiments involving mechanical forces appear to indicate that the isolated mechanical influence on cells and tissues may not be sufficient for efficient microtubule reorientations. Returning to the previously discussed bending-related transverse reorientations in isolated coleoptiles, these mechanically triggered reorientations did not occur as readily in auxin-free coleoptile segments as when auxin was externally supplied [1]. Therefore, while reorientations were indeed triggered by mechanical extension or compression, it appears that an additional condition, provided by auxin, greatly facilitated them. In light of our data, we think it is likely that this facilitating condition relates to the ‘growth activation status’ of the cell, discussed above, rather than to auxin signaling specifically. Similarly, Burian and Hejnowicz attempted to trigger reorientations by stretching isolated peels of sunflower epidermis with externally attached weights [20,21]. Even though the peels were elongating under the applied force, transverse reorientations were not observed. However, reorientation did happen if fusicoccin was applied to the peels, which presumably changed the physiological condition of this isolated, auxin-depleted tissue into a growth-like state.

Thus, while physical forces acting in cell walls most likely participate in microtubule reorientation, the mechanical influences may not be sufficient by themselves. A particular ‘physiological condition for growth’ in vivo provided by auxin and experimentally by either auxin or fusicoccin (Figure 6, [21])—and which may include turgor changes (Figure 5), the modulation of the physical properties of cell walls according to the acid growth model, and possibly also other aspects of cell physiology—constitutes a likely part of the optimal reorientation trigger.

Finally, as a side observation, one of our experiments showed that the signaling for reorientation originating from growth does not involve the biosynthesis of a growth-reactive proteinaceous reorientation factor. Microtubule reorientation by fusicoccin was possible when protein translation was inhibited by cycloheximide (Figure 6C). However, there could exist a reorientation factor which is expressed constitutively but only activated in response to growth—by phosphorylation, for instance. In an interesting contrast, Elliott and Shaw recently proposed that transverse microtubule array reorientation does require the expression of a protein factor [44] whose role is, however, limited to the suppression of longitudinal microtubules, while it does not act in instructing transverse microtubules themselves.

### 3.3. Auxin-Mediated Inhibition of Root Growth and Longitudinal Microtubule Reorientation

We wish to point out that the present study specifically focused on the hypocotyl, where auxin causes promotion of growth, which leads in turn to transverse microtubule reorientation. In contrast, the causal relationship of analogical but opposite phenomena in the root was not addressed here. In the root, high auxin signaling leads to growth inhibition, rather than promotion, and to the associated reorientation of microtubule arrays into longitudinal, rather than transverse, arrays. Formerly, Laskowki proposed that the longitudinal reorientation of microtubules follows growth rate decline, but that it must be triggered by factors distinct from the change of elongation rate [45]. More recently, the idea that a direct and rapid longitudinal reorientation of microtubules by auxin is part of the mechanism by which auxin inhibits growth (akin to Hypothesis A, recall Figure 1) has been a matter of debate [8,46,47]. Based on this recent discussion and on the findings presented here, we recognize that the reorientation of microtubules into longitudinal arrays is not likely to be a direct prerequisite for inhibition of root cell elongation by auxin. Root growth inhibition by high auxin levels likely occurs without the involvement of microtubules. Regarding the direct cause of longitudinal microtubule reorientations in the root during growth inhibition, we may speculate that, in analogy to Hypothesis C, microtubules in the root do not react to auxin signaling but only to growth changes. However, we feel that, at present, we do not have grounds to dismiss a Hypothesis B-type model in which the longitudinal reorientation of microtubules is in fact triggered by a specific branch of auxin signaling, while another independent but temporally correlated branch signals for root growth inhibition. This question appears particularly uncertain in light of the recent finding that, differently to the hypocotyl, the auxin control of root growth is mediated through a novel and not well understood non-transcriptional branch of SCF^TIR1/AFB^ signaling [48].

## 4. Materials and Methods

### 4.1. Plant Material

The following previously described lines were used in this study: *35S:GFP-MAP4* [28], *HS:axr3-1* [27], and *DR5:Luciferase* [49]. Experiments involving axr3-1 were performed in the F1 generation of *HS:axr3-1* x *35S:GFP-MAP4*.

### 4.2. Seedling Growth

Seedlings of *Arabidopsis thaliana* (L.) Heynh. were grown on a ½MS (Murashige and Skoog) medium with 1% (*w*/*v*) sucrose at 21 °C in darkness for three days, after an initial period of light exposure to promote seed germination.

### 4.3. Hypocotyl Handling and Experimental Design

Hypocotyls were separated from roots and cotyledons by razor blades. Cuts were made at or just below the apical hook and at the root–shoot junction. During the course of experiments, seedlings were placed on a solid ½MS medium with 1% (*w*/*v*) sucrose, which was supplemented with necessary chemicals for treatments. The following chemicals were used as treatments: Indole-3-acetic acid, mannitol, fusicoccin, and cycloheximide. Potential pH changes in supplemented media were not controlled.

To avoid a possible influence of red and blue light on microtubule array orientations, hypocotyls were manipulated under weak green light and otherwise kept in darkness up until the point of live imaging. Any potential remaining effect of light would have been identical for all conditions tested and therefore does not constitute an uncontrolled experimental variable.

At least three repetitions of each microtubule orientation experiment were performed, with approximately 8–16 hypocotyls, corresponding typically to 50–100 cells, used for each condition/genotype in each experiment. Details for each experiment are given in figure legends. Samples were typically processed in batches of 8. This batched processing included the isolation of hypocotyls by razor blades, the transference to treatment media, and imaging. Thus, the timing of auxin depletion after hypocotyl isolation and the timing of treatments leading up to imaging were consistent and precise. In case of experiments with a larger total sample size, the distinct treatments or genotypes were alternated in consecutive batches. All these precautions were intended to remove the possible confounding influences of environmental conditions and to equalize any possible side effects of wounding caused by cutting, as well as stresses associated with hypocotyl isolation.

End-point array orientations were compared between treatments or genotypes, while no comparison was made with the initial state before treatments. The large sample sizes used assure that, on average, the initial condition of microtubule arrays was the same in all cases (that is, the auxin-depleted condition of mainly longitudinal microtubules) and, as such, did not need to be specifically monitored in each experiment.

We noticed a certain degree of variability between experiments in the effect of the basic treatment (1 h of 10 μM IAA) in terms of the average degree of microtubule reorientation. Though 3-day-old seedlings were always used, the variability appeared to correlate with the exact age of the seedlings and, therefore, with hypocotyl size, in that a slightly younger and shorter hypocotyls reacted better. However, in each single experiment, the seedlings used were grown together, and therefore were of exactly the same age and very similar size. Experimental conclusions were drawn from side-by-side comparisons within each experiment rather than from quantitative comparisons between experiments.

For experiments involving cycloheximide, a depletion medium [22] was used instead of the ½MS medium with 1% (*w*/*v*) sucrose, because, in comparison to a slight and delayed inhibitory effect of cycloheximide on fusicoccin-induced growth on the depletion medium [23], a stronger growth-inhibitory effect of cycloheximide was observed on the standard medium, which interfered with correct experimentation.

*HS:axr3-1* x *35S:GFP-MAP4* seedlings were induced by a 40-min incubation at 37 °C together with *35S:GFP-MAP4* controls, approximately 3 hours prior to cutting of hypocotyls.

### 4.4. Imaging and Scoring of Cortical Microtubule Arrays

*35S:GFP-MAP4* hypocotyls were imaged using a Zeiss LSM700 confocal microscope with a 20× lens. Microtubules were imaged in a sub-apical region of the hypocotyls, where, at the stage of development used, rapid cell elongation is possible. For microtubule orientation assessment, microscopic pictures from all treatment conditions or genotypes of a single experiment were collected together, randomized by a computer script (Supplementary materials), and then scored blindly without explicit knowledge of the condition or genotype. Each cell was classified as having a longitudinal, oblique, transverse, or random microtubule array. Column graphs were prepared in Microsoft Excel and represent the mean frequencies of orientation classes between experiments. Statistical evaluation was done using *t* tests on frequencies of longitudinal arrays. Longitudinal arrays were chosen for simplicity, because microtubule array reorientations resulted in a gross change from this single class into two equally relevant classes: Oblique and transverse.

For the control experiment on microtubule dynamics in mannitol-treated cells, time lapses of 20 images taken every 5 s were captured with 40× lens. A total of 39 movies per treatment from around 15 hypocotyls were captured in the course of three repeated experiments.

### 4.5. Hypocotyl Growth Measurements

Wild-type Col-0 seedlings were used for growth experiments. Seedlings were handled as for microtubule imaging experiments. The growth of isolated hypocotyl segments was captured using a flatbed office scanner, with hypocotyls placed in small Petri dishes on a solid ½MS medium with 1% (*w*/*v*) sucrose supplemented with treatment chemicals. Hypocotyl length before and after a 1-hour growth period was measured using ImageJ and expressed as ratio. Dot plots (stripcharts) were prepared with RStudio. The normal growth reaction of *35S:GFP-MAP4* to applied auxin was additionally confirmed.

### 4.6. Auxin Signaling Evaluation

Hypocotyls were isolated from dark-grown seedlings of the *DR5:Luciferase* line, and during the depletion of internal auxin, incubated for 30 min in drops of 1 mM d-luciferin in a 1× PBS buffer on a depletion medium [22]. Samples were then transferred to media supplemented with IAA alone or IAA and mannitol in multi-well tissue culture plates. These were transferred to a dark box, and luminescence was captured using a Photometrics Evolve 512 EMCCD camera equipped with a 17 mm fixed lens/0.95 and an additional 125 mm lens. The EMCCD multiplier gain was set to 150 and the exposure time to 110 s.

## 5. Conclusions

The organization of microtubules into ordered arrays beneath the plasma membrane and the growth-related reorientations of these arrays in elongated cells of root and stem epidermis have fascinated plant biologists for a long time. In this work, we revisited the question of the exact causes of reorientations in hypocotyl epidermis with a new, comprehensive set of experiments in *A. thaliana*, and we attempted to synthesize various previously published results on related topics. We reach the following conclusion: It is not auxin itself that causes microtubule reorientation—meaning that there is no distinct signaling branch of the SCF^TIR1/AFB^-ARF-Aux/IAA pathway that leads to reorientation. Reorientation in elongating hypocotyl epidermal cells results from the cell growth process, which is, indeed, controlled by auxin in vivo but, experimentally, can be triggered without auxin. This growth-related reorientation may be connected to turgor changes as well as to physical forces acting in cell walls, although it is not yet exactly clear how. We hope that these conclusions will prove to be a useful contribution to future research on cortical microtubules.

## Supplementary Materials

Supplementary materials can be found at https://www.mdpi.com/1422-0067/20/13/3337/s1.

## Abbreviations

AHA	autoinhibited H^+^-ATPase
ARF	auxin response factor
Aux/IAA	auxin/indole-3-acetic acid
CHX	cycloheximide
CLSM	confocal laser scanning microscopy
FC	fusicoccin
GFP	Green fluorescent protein
IAA	Indole-3-acetic acid
MAP4	Microtubule-associated protein 4
MS	Murashige and Skoog
SAUR	small auxin up RNA
TIR1/AFB	transport inhibitor response 1/auxin signaling F-box protein
